# The Diagnostic and Prognostic Value of ^18^F-FDG PET/MR in Hypopharyngeal Cancer

**DOI:** 10.3390/diagnostics15172119

**Published:** 2025-08-22

**Authors:** Cui Fan, Xinyun Huang, Hao Wang, Haixia Hu, Jichang Wu, Xiangwan Miao, Yuenan Liu, Mingliang Xiang, Nijun Chen, Bin Ye

**Affiliations:** 1 Department of Otolaryngology & Head and Neck Surgery, Ruijin Hospital, Shanghai Jiao Tong University School of Medicine, No. 197 Ruijin 2nd Road, Shanghai 200025, China; fcdoc_022@126.com (C.F.); mingliangxiang@163.com (M.X.); 2 Shanghai Key Laboratory of Translational Medicine on Ear and Nose Diseases, Shanghai 200125, China; 3 Department of Nuclear Medicine, Ruijin Hospital, Shanghai Jiao Tong University School of Medicine, Shanghai 200025, China

**Keywords:** ^18^F-FDG PET/MR, hypopharyngeal cancer, TNM staging, larynx preservation, prognosis

## Abstract

**Objective:** To evaluate the diagnostic performance of fluorine 18 fluorodeoxyglucose positron emission tomography/magnetic resonance imaging (^18^F-FDG PET/MR) in the preoperative staging of hypopharyngeal cancer (HPC), compare it with conventional enhanced computed tomography (CT) and MR, and further explore the prognostic value of its metabolic and diffusion metrics for HPC. **Methods:** This retrospective study included 33 patients with pathologically confirmed HPC. All patients underwent preoperative ^18^F-FDG PET/MR, CT, and MR examination. The staging performance of the three modalities was evaluated using pathological staging as a reference. Additionally, metabolic indicators and diffusion-related parameters from PET/MR were collected to investigate their impact on larynx preservation and survival. **Results:** PET/MR demonstrated accuracies of 90.9% and 71.4% in the preoperative T and N staging, respectively, significantly higher than those of CT (54.5%, *p* = 0.001; 42.9%, *p* = 0.021) and MR (66.7%, *p* = 0.016; 42.9%, *p* = 0.021). On the whole, significant differences emerged in the maximum standard uptake value (SUVmax), metabolic tumor volume (MTV), minimum apparent diffusion coefficient (ADCmin), and mean ADC (ADCmean) and combined ratios across different T stages, while SUVmax, mean SUV (SUVmean), total lesion glycolysis (TLG), and MTV varied significantly across different N stages. The ADCmin and ADCmean showed good predictive capability for larynx preservation, with AUCs of 0.857 and 0.920 (*p* < 0.05), respectively. In Cox multivariate analysis of overall survival, high-level ADCmean (*p =* 0.004) and low-level TLG/ADCmean (*p* = 0.022) were significantly associated with better survival. **Conclusion:** In HPC, ^18^F-FDG PET/MR imaging significantly surpasses CT and MR in preoperative diagnostic staging. Its diffusion-related parameters have substantial prognostic value, with high ADC values associated with larynx preservation. ADCmean and TLG/ADCmean are potential prognostic indicators for HPC.

## 1. Introduction

Hypopharyngeal cancer (HPC) exhibits the poorest prognosis among head and neck cancers (HNCs), with a five-year survival rate ranging from 25% to 50% [1,2]. Owing to its hidden location and atypical early symptoms, many patients are diagnosed at advanced stages [3]. The hypopharynx occupies a unique anatomical position at the junction of the pharynx and larynx, forming the upper esophageal boundary [4,5]. In comparison to other HNCs, HPC demonstrates a pronounced tendency for lymph node metastasis, even with a small primary lesion [6].

Presently, enhanced computed tomography (CT) and magnetic resonance (MR) imaging are the predominant modalities employed in the preoperative evaluation of HPC. CT imaging is time-efficient, cost-effective, and offers clear anatomical structures. MR excels in visualizing soft tissues and provides superior evaluation of cartilage invasion and submucosal spread with reduced radiation exposure [7,8]. Furthermore, positron emission tomography (PET)-CT is progressively employed in HPC, primarily due to its effectiveness in identifying lymph node and distant metastases [9,10]. PET/MR is valued for its superior tissue resolution and minimized radiation exposure. PET/MR combines metabolic data with advanced MR sequences—including spectroscopy, diffusion-weighted imaging (DWI), and functional imaging—offering enhanced lesion characterization without the radiation burden of PET/CT [11].

The hypopharynx is encompassed by diverse cartilaginous tissues, which offer distinct advantages for PET/MR imaging utilization. Recently, PET/MR matches or surpasses PET/CT in evaluating primary and metastatic sites in HNCs [12,13]. However, due to its relatively rare occurrence, few studies have examined PET/MR’s utility in HPC. Our study aims to compare the diagnostic efficacy of ^18^F-FDG PET/MR with conventional CT/MR for clinical staging of HPC and to preliminarily assess the significance of ^18^F-FDG PET/MR parameters in preserving laryngeal function and enhancing survival rates.

## 2. Materials and Methods

### 2.1. Patients

This study was approved by the ethical committee of our institution. Between July 2019 and November 2022, we recruited 34 patients who were initially diagnosed with HPC at our institute and confirmed by pathology. Patients were included if they met the following criteria: (1) initial diagnosis at our institution; (2) pathological confirmation of HPC; (3) underwent preoperative laryngeal CT and MR, as well as whole-body ^18^F-FDG PET/MR examinations; (4) underwent primary lesion resection and cervical lymph node dissection. Patients were excluded if they had: (1) a history of malignant tumors; (2) non-squamous cell carcinomas of the hypopharynx; (3) uncontrolled hyperglycemia; (4) preoperative radiotherapy, chemotherapy or immunotherapy. Patient information is collected through medical record retrieval or telephone follow-up, with a follow-up schedule of every 3 months for the first 2 years post-treatment, then every 6 months until 5 years post-treatment, and annually thereafter. Regular endoscopic and imaging examinations are performed during follow-up periods. Overall survival (OS) is defined as the time from examination date to death or last follow-up, with the study closure date set for 30 December 2024.

Staging for primary tumors and lymph nodes is based on AJCC (version 8). Intraoperative findings and surgical pathology serve as reference standards for TNM staging.

### 2.2. ^18^F-FDG PET/MR

The examinations were conducted using an integrated PET/MR system (Biograph mMR; Siemens, Erlangen, Germany). Patients received an intravenous injection of ^18^F-FDG (2–5 MBq/kg). The PET/MR scan comprised two components: a whole-body scan and a detailed neck scan. The former encompassed imaging from the top of the skull to the upper third of the thigh. The latter scanned from the mandible to the supraclavicular fossa, acquiring 1 mm thick cross-sectional and coronal images. Simultaneous collection of PET and MR images occurred, with MR sequences consisting of T2WI-HASTE high-throughput sequences, T2WI-BLADE sequences, T1W1 sequences, T1WI-DIXON sequences, DWI sequences, and T1WI starvibe. ADC values were derived using a single exponential function with b values of 50 and 800 s/mm^2^.

The identification of ^18^F-FDG positive lesions was carried out by two seasoned nuclear medicine physicians. The original images were exported in DICOM format and processed using RadiAnt DICOM Viewer software (version 2021.1). Observers were assigned DICOM data randomly without any medical history. ^18^F-FDG positive lesions were identified based on uptake intensities surpassing anticipated physiological levels. MR positive lesions were delineated based on experience, with a consensus achieved through discussion. They manually outlined the tumor boundaries at the largest part of the target lesions to define the region of interest (ROI) for each lesion. The software automatically computed the maximum standardized uptake value (SUVmax), mean SUV (SUVmean), total lesion glycolysis (TLG), metabolic tumor volume (MTV) on MR images, and minimum apparent diffusion coefficient (ADCmin) as well as mean ADC (ADCmean). MTV was calculated using 40% of SUVmax as the threshold, while TLG was calculated as the product of MTV and SUVmean.

### 2.3. CT and MR

CT scans were performed using scanners (Siemens, Erlangen, Germany), with the administration of an iodine contrast agent 70 s before scanning. Scanning encompassed from the base of the skull to the supraclavicular fossa, with axial sequences having a 1 mm slice thickness and coronal sequences a 3 mm thickness.

All patients underwent scanning with a 3.0 T MRI scanner (Ingenia, Philips Healthcare, Eindhoven, The Netherlands), both prior to and following the injection of gadolinium DTPA contrast agent. The scanning range extended from the top of the temporal lobe to the supraclavicular fossa. The acquired images, both axial and coronal, had a slice thickness of 5 mm and an interslice distance of 1 mm.

CT and MR images were managed via the PACS system and randomly assigned to two radiologists for analysis. In cases of disagreement, discussions were conducted to reach a consensus, with no reference to clinical or other imaging data during the review.

### 2.4. Statistical Analysis

The normality of the data was assessed using the Kolmogorov–Smirnov test. Continuous variables were compared using independent-samples *t*-tests (Mann–Whitney test for non-parametric data) with a two-tailed probability. Categorical variables were evaluated using the paired Chi-square test (McNemar’s test). Differences between the means of multiple groups were analyzed using one-way ANOVA (Kruskal–Wallis ANOVA for non-parametric data) with Bonferroni post-hoc comparison. The Spearman correlation coefficient was calculated to examine the relationship between two variables. Univariate and multivariate Cox-regression analysis was performed to assess the effect of significant parameters. Receiver Operating Characteristic (ROC) analysis was performed to evaluate diagnostic performance. The Kaplan–Meier method and the log-rank test were used to assess the difference in OS between groups, and the median values of continuous variables were used as the cut-off values. A *p*-value < 0.05 was considered statistically significant. Data analysis was carried out using IBM SPSS Statistics (version 22.0), and the results were visualized using GraphPad Prism (version 9.0).

## 3. Results

### 3.1. Patient Characteristics

The study cohort comprised 33 male patients diagnosed with HPC, with an average age of 62.7 years (Table 1). Primary tumors occurred most frequently in the pyriform sinus (22 cases), followed by postcricoid area (6 cases), and posterior pharyngeal wall (5 cases). Tumor differentiation revealed that 19 cases (57.6%) were moderately differentiated, 13 cases (39.4%) were poorly differentiated, and only 1 case (3.0%) was well-differentiated. Regarding primary lesion staging, 16 cases (48.5%) were classified as T3, 11 cases (33.3%) as T4, and 6 cases (18.2%) as T2. Lymph node metastasis was observed in 26 patients (78.5%), with 11 cases each of N2 and N3 (33.3%), and fewer cases of N1 (4 cases, 12.1%). As for TNM stage, there were 23 cases (69.7%) with stage IV, 8 cases (24.2%) with stage III, and only 2 cases (6.1%) with stage II. Local advanced stage cases accounted for 93.9%. Notably, laryngeal function was preserved in 25 patients (75.8%). Throughout the follow-up period, five patients (15.2%) experienced recurrence, while nine patients (27.3%) died.

### 3.2. Comparison of Clinical Staging for HPC Using PET/MR, CT, and MR

Clinical staging plays a pivotal role in surgical decision-making and prognosis for HPC. In this study, the efficacy of three methods in assessing clinical staging was compared with pathological staging as the gold standard (Table 2). The data revealed that PET/MR exhibited an overall accuracy of 90.9% in preoperative T staging, which was significantly higher than MR (66.7%, *p* = 0.016) and CT (54.5%, *p* = 0.001). There was no significant difference between T staging of MR and CT. When examining specific T staging, PET/MR was notably superior in assessing T3 compared to CT (87.5% vs. 43.8%, *p* = 0.016). In terms of N staging, PET/MR demonstrated excellent accuracy (90.9%), significantly outperforming both MR and CT (66.7%, *p* = 0.021). Although PET/MR consistently showed higher accuracy across different N stages, no statistical differences were found. PET/MR demonstrated significantly higher accuracy than both CT (90.9% vs. 66.7%, *p* = 0.008) and MR (90.9% vs. 69.7%, *p* = 0.016) in clinical TNM staging, similar to the results obtained for T staging. Overall, PET/MR exhibited prominently higher diagnostic efficacy for clinical staging compared to CT and MR. MR performed better than CT, though with no significant difference.

### 3.3. Correlation of PET/MR Parameters with Clinical Staging

One advantage of PET/MR imaging lies in its capacity to offer a comprehensive evaluation of tumor characteristics. This study specifically delves into the assessment of tumor metabolic markers (SUVmax, SUVmean, TLG, MTV), cellular diffusion indicators (ADCmean, ADCmin) and combined parameters of PET/MR, examining their associations with clinical staging (Table 3 and Table 4).

**Figure 1:**
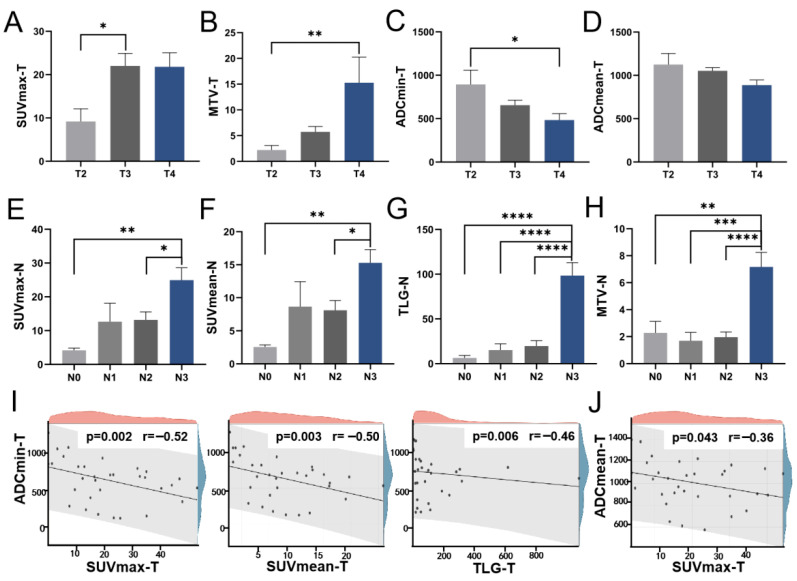
The distribution of PET/MR parameters across T and N stages. The levels of primary lesion SUVmax (**A**), MTV (**B**), ADCmin (**C**), and ADCmean (**D**) at various T stages; The levels of lymph node SUVmax (**E**), SUVmean (**F**), TLG (**G**), and MTV (**H**) across different N stages; (**I**) The scatter diagram illustrating the correlations of primary lesion ADCmin with SUVmax, SUVmean, and TLG, respectively; (**J**) The scatter diagram illustrating the correlation between primary lesion ADCmean and SUVmax. SUV Standardized uptake value, TLG Total lesion glycolysis, MTV Metabolic tumor volume, ADC Apparent diffusion coefficient. * *p* < 0.05, ** *p* < 0.01, *** *p* < 0.001, **** *p* < 0.0001.

Our statistical analysis revealed notable variations in SUVmax, MTV, ADCmin, and ADCmean of the primary tumor across distinct T stages. Notably, SUVmax exhibited a significant discrepancy between the T2 and T3 groups (Figure 1A). MTV demonstrated a progressive increase with advancing T staging, while ADCmin exhibited a corresponding decrease, with both parameters showing significant disparities between the T2 and T4 groups (Figure 1B,C). Although ADCmean displayed variations across T stages, the differences did not reach statistical significance between specific groups (Figure 1D). Furthermore, we conducted an assessment of the relationship between metabolic and diffusion indices of the primary lesion, uncovering a discernible negative correlation, notably between ADCmin and SUVmax (r = −0.52, *p* = 0.002) (Figure 1I,J). Interestingly, our analysis revealed significant differences in combined parameter indices across T-staging levels, with values increasing progressively with advancing T-stage. The distribution differences for these combined indices demonstrated greater statistical significance than those observed for individual metabolic parameters, particularly for SUVmax/ADCmean, TLG/ADCmean, and MTV/ADCmin. We also compared the differences in these parameters among different TNM stages (Table S1). Although SUVmax, SUVmean, MTV, and TLG increased with advancing stages, the differences were not statistically significant. No significant differences were observed in other parameters or combined parameters either.

We then explored the variability of these indices across N stages. Grouped by N staging, significant differences were observed in the lymph node indices of SUVmax, SUVmean, TLG, and MTV, particularly in TLG and MTV. Detailed comparisons revealed marked differences in SUVmax and SUVmean between N0 and N3, and between N2 and N3 (Figure 1E,F). Similarly, TLG and MTV exhibited significant variations among four groups (Figure 1G,H). Conversely, ADC-related metrics showed no significant variance across N stages, suggesting that metabolic indices provide more relevant insights for N staging assessment.

### 3.4. Correlation Between PET/MR Parameters and Prognosis

**Figure 2:**
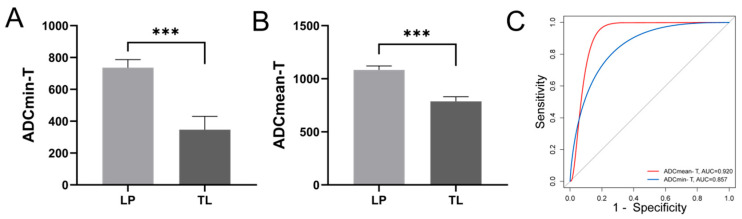
Relationship between primary lesion diffusion parameters and laryngeal preservation in patients with HPC. The differences in primary lesion ADCmin (**A**) and ADCmean (**B**) between LP and TL groups; (**C**) The ROC curves for larynx preservation prediction using primary lesion ADCmin and ADCmean, AUC-ADCmin-T = 0.857, *p* = 0.003, AUC-ADCmean-T = 0.920, *p* < 0.001. ADC Apparent diffusion coefficient, LP Larynx-preserving, TL Total laryngectomy, AUC Area under the curve. *** *p* < 0.001.

Preserving laryngeal function is essential for the quality of life in patients with HPC. We investigated the relationship between PET/MR parameters and both laryngeal preservation and survival outcomes in HPC patients. The cohort comprised two groups: those who underwent larynx-preserving treatment (LP) and those receiving total laryngectomy (TL). We found that the diffusion indices ADCmin and ADCmean of the primary tumor were significantly lower in the TL group (Figure 2A,B). However, the metabolic indices of the primary tumor did not display significant differences. Furthermore, we analyzed the predictive ability of PET/MR parameters for larynx preservation using ROC curves, which showed that ADCmin and ADCmean had AUCs of 0.857 (*p* = 0.003) and 0.920 (*p* < 0.001), respectively, both demonstrating good predictive effectiveness (Figure 2C). Patients with higher ADCmin (>529.5) and ADCmean (>921.5) demonstrated higher rates of laryngeal function preservation.

**Figure 3:**
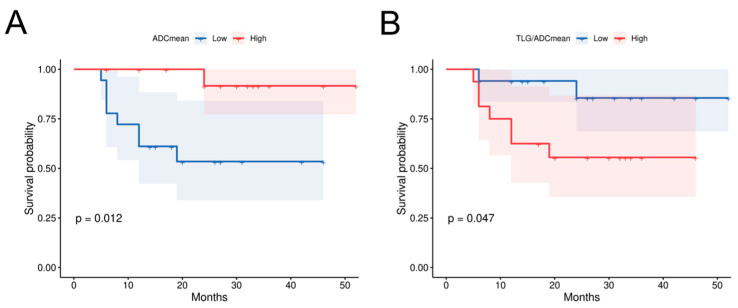
Correlation between primary lesion diffusion parameters and survival in HPC patients. (**A**) The prognostic survival curve for HPC using primary lesion ADCmean, *p* = 0.012; (**B**) The prognostic survival curve using primary lesion TLG/ADCmean, *p =* 0.047. TLG Total lesion glycolysis, ADC Apparent diffusion coefficient.

To identify the prognostic factors for OS in HPC, we first conducted univariate Cox analysis on multiple variables including age, T stage, N stage, TNM stage, pathological grade, and PET/MR parameters and their ratios (Table 5). The results showed that ADCmean (*p* = 0.007), ADCmin (*p* = 0.029), SUVmax/ADCmean (*p* = 0.028), MTV/ADCmean (*p* = 0.037), TLG/ADCmean (*p* = 0.021), MTV/ADCmin (*p* = 0.03), and TLG/ADCmin (*p* = 0.021) were all significant factors, which subsequently underwent multivariate analysis. ADCmean (HR 0.995, 95% CI 0.992–0.998, *p*  =  0.004) and TLG/ADCmean (HR 1.002, 95% CI 1.001–1.008, *p*  =  0.022) emerged as significant prognostic factors for HPC survival after multivariate analysis. Using the median values of these two indicators as cut-off values, we analyzed the differences between groups through KM curves and log-rank tests (Figure 3). Patients of HPC with lower ADCmean values (1046) or higher TLG/ADCmean ratios (52.5) demonstrated significantly worse survival outcomes.

### 3.5. CT, MR, and PET/MR Imaging Comparison

**Figure 4:**
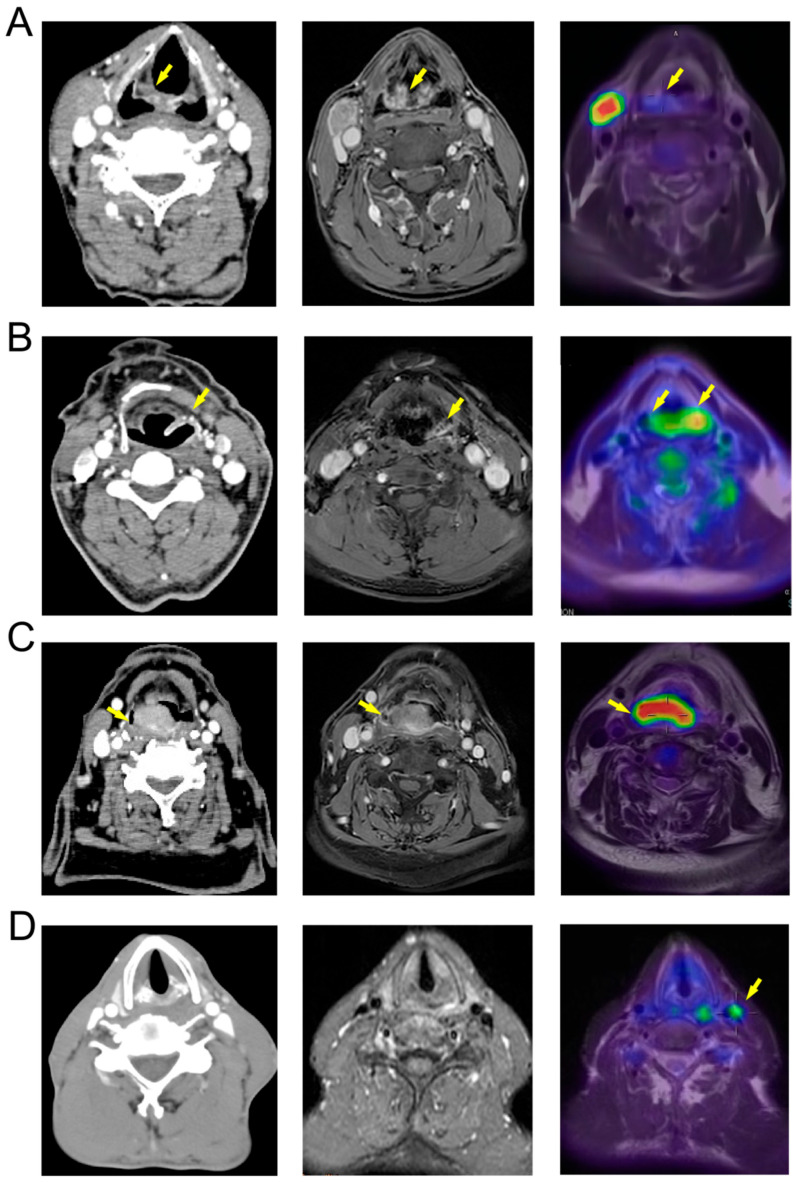
Comparison of CT, MR, and PET/MR images of HPC. (**A**) The primary lesion (yellow arrow) is located on the medial wall of the right piriform sinus (T1) and is difficult to distinguish in CT and MR images, but shows increased metabolism in PET/MR; (**B**) The primary lesion (yellow arrow) is located on the medial, lateral, and posterior walls of the left hypopharynx (T3), appearing more localized in CT and MR images, while PET/MR shows a high-metabolism lesion extending beyond the midline, consistent with intraoperative findings; (**C**) The primary lesion (yellow arrow) is located on the posterior wall of the pharynx (T3), with unclear boundaries in CT and MR images and suspected involvement of the prevertebral fascia, while PET/MR indicates that the tumor does not breach the prevertebral fascia; (**D**) The patient’s primary lesion is located in the left piriform sinus (T3), with no obvious positive lymph nodes shown in CT and MR, but PET/MR indicates high metabolism in the left cervical sheath lymph nodes (yellow arrow), with postoperative pathology indicating positivity.

We conducted a comparative analysis of CT, MR, and PET/MR imaging techniques, demonstrating that PET/MR offers substantial benefits in assessing HPC, which frequently presents as a mucosal diffusion type. Traditional CT and MR imaging are less effective in delineating the complete mucosal thickening. For instance, in Figure 4A, laryngoscopy identified the primary lesion on the inner wall of the left piriform fossa. However, CT and MR missed this finding. In contrast, PET/MR imaging detected increased metabolic activity in the right hypopharyngeal region. Similarly, in the case depicted in Figure 4B, CT and MR imaging showed localized thickening and enhancement of the left laryngopharyngeal sidewall, suggesting a T2 stage. However, PET/MR imaging revealed midline-crossing extension exceeding 4 cm, prompting reclassification to T3—later verified by endoscopic and surgical findings.

For HPC in the posterior pharyngeal wall, assessing whether the lesion extends into the prevertebral fascia is critical for surgical planning. In Figure 4C, the CT scan revealed a lesion of posterior pharyngeal wall with pronounced enhancement and indistinct margins, complicating the evaluation of the prevertebral fascia. Conversely, the MR images (T1 enhanced phase) provided a clearer delineation of the lesion, showing intact intervertebral fat spaces. PET/MR imaging further clarified the extent of the metabolically active lesion, confirming its non-involvement of the prevertebral fascia. PET/MR also proves essential in detecting metastatic lymph nodes. In the case of Figure 4D, while CT and MR scans did not identify any suspicious lymph nodes based on morphology, size, and enhancement, PET/MR identified highly metabolic lymph nodes adjacent to the left cervical sheath. Consequently, the patient underwent left functional neck dissection. Postoperative pathology confirmed a metastatic lymph node in the left cervical zone III.

## 4. Discussion

PET/MR imaging has garnered considerable attention since its introduction, primarily owing to its low radiation exposure and superior soft tissue visualization. However, the adoption of this technology has been tempered by its high costs and prolonged acquisition times. Studies have demonstrated the significant value of PET/MR in the staging and treatment monitoring of malignancies such as breast and ovarian cancer [14,15,16,17]. Here, we present the first dedicated examination of HPC using ^18^F-FDG PET/MR, assessing both its diagnostic and prognostic capabilities and comparing its effectiveness to that of CT and MR imaging.

In assessing T staging, PET/MR consistently demonstrated superior accuracy over both CT and MR, likely due to the unique anatomical location and three-dimensional growth pattern of HPC. In superficial infiltration cases, neither CT nor MR effectively enhances the image, complicating the staging process. The superior soft tissue resolution of MR proved particularly valuable for evaluating cartilage invasion. For N staging, PET/MR also outperformed both CT and MR, particularly in distinguishing between N0 and N1 stages. While CT and MR depend predominantly on lymph node morphology and dimensions for evaluation, some N1 stage lymph nodes do not show notable differences on these imaging modalities. In such cases, the metabolic intensity provided by PET/MR serves as a crucial diagnostic tool.

As an indicator of tissue water mobility, ADC has been found to have an inverse relationship with SUV in cancers such as breast and cervical cancer [18,19,20]. Despite a few controversies, the majority of studies in HNC support this relationship between metaboli and diffusion markers. For example, Nakajo M et al. studied PET/CT and DWI in HNC and found a significant negative correlation between SUVmax and ADC of primary tumors, suggesting that they have similar potential in predicting the survival of HNC patients [21]. Wongsa P et al. reported a strong and negative correlation between SUVmax and the median ADV (r = −0.75, *p* = 0.01) of PET/MR in HNC [22]. Zhang L et al. investigated integrated cervical PET-MR parameters of HPCs and reported a moderate negative correlation between ADCmean and MTV [23]. In our study, we observed a significant negative correlation between primary lesion ADCmin and SUVmax of HPC. Regarding Tstaging, ADCmin and MTV values significantly variated as the T stage progressed, with more pronounced distribution differences than those seen with SUVmax. Both ADCmin and MTV have been previously reported to be significantly associated with T staging in cervical cancer [24]. This also demonstrates the high evaluative value of ADC values for evaluating primary lesions of HPC. In N staging, the findings show significant differences in metabolic markers, particularly TLG. TLG increases with advancing N stages, showing marked differences. TLG has been reported to correlate with N staging in colorectal cancer, while its role in HPC is yet to be clarified [25]. The difference in ADC distribution between T and N staging may be linked to a higher proportion of necrosis in metastatic lymph nodes in HPC. In our study, nearly half of the cases with lymph node metastasis presented with lymph node necrosis.

This research initially investigates the prognostic value of whole-body PET/MR for laryngeal preservation and survival outcomes in HPC. We discovered that high-level ADCmean was significantly associated with laryngeal preservation and better survival outcomes, serving as a crucial prognostic factor. ADC retrieved from PET/MR shows outstanding prognostic value, superior to metabolic markers such as SUV and TLG, related to its histological significance in tumors. Huang C et al. examined cervical regional PET-MR results from 22 HPC patients [26]. In their findings, ADCmean was also an effective predictor of OS in univariate analysis, although it did not show significance in multivariate analysis. Recently, Boeke S et al. explored multifunctional combined PET/MR on xenograft HNC mouse models, and proposed that ADC values correlate with radiotherapy sensitivity under multidimensional imaging, offering potential prognostic value for HNC [27]. Hans-Jonas Meyer et al. have reported that high ADC values correlate with a higher proportion of tumor-infiltrating lymphocytes (TILs) in HNC, which also serve as independent prognostic markers for HNC [28]. This study underscores the importance of diffusion indices over metabolic indices in the prognosis evaluation of HPC.

Recent studies have demonstrated the prognostic value of combined PET/MRI parameters in malignant tumors. The study of Kim YI et al. proposed that MTV and combined PET/MRI parameters including SUVmax/ADCmean, MTV/ADCmean, and TLG/ADCmean could predict treatment failure of HNC patients after surgery [29]. Pace L et al. identified SULpeak and SULpeak/ADCmean as significant predictors of OS in patients with locally advanced oropharyngeal and hypopharyngeal cancers undergoing chemoradiotherapy [30]. Consistent with previous findings, our study indicates that SUVmax/ADCmean, MTV/ADCmean, and TLG/ADCmean not only exhibit significant differences across different T stages of HPC but also serve as effective predictors of OS, performing better than individual metabolic parameters. Among these, TLG/ADCmean demonstrates particularly strong performance in both tumor staging and prognostic assessment.

The hypopharynx sits at the anatomical junction of the larynx, pharynx, and esophagus. The tumor’s proximity to structures such as the thyroid cartilage, cricoid cartilage, and prevertebral space significantly influences surgical planning. PET/MR integrates metabolic data with MR imaging, improving lesion detection, particularly in tumors with infiltrative and diffuse growth patterns. Our comparative analysis indicates that PET/MR more accurately assesses tumor spread in these cases, which are typically responsive to neoadjuvant chemotherapy. This underscores the potential of PET/MR in monitoring the effectiveness of such treatments in HPC. According to Huang et al., PET/MR outperforms PET-CT, standalone MR, and CT in detecting invasion into the masseter muscle space in advanced buccal squamous cell carcinoma [31]. In HPC, the combination of MR and PET has also been reported to improve the diagnostic accuracy for determining the invasion of a tumor into the prevertebral space [32]. The MR components of PET/MR utilize thin-layer sequences centered on the tumor, offering detailed insights into both the tumor and adjacent structures. Combined metabolic and structural data provide comprehensive delineation of tumor boundaries.

Our study analyzed the diagnostic and prognostic value of ^18^F-FDG PET/MR in HPC, providing preliminary evidence to support its clinical application. We found that PET/MR significantly outperforms CT and MR in the accuracy of T staging and N staging of HPC. Its parameters also show significant differences across different stages, particularly SUVmax and SUVmean. Diffusion-related indices correlate more closely with prognosis: lower ADC values indicate worse survival in HPC, especially lower ADCmean values. Moreover, the combined PET/MR parameters increased significantly with advancing T-stages, demonstrating higher significance than individual parameters, particularly the TLG/ADCmean. The latter was also identified as an independent prognostic factor for HPC. However, several limitations warrant consideration, including the modest sample size, which constrains the generalizability of our findings. As PET/MR becomes more widely used, more cases can be included in future analyses. Additionally, this study lacks an analysis of the correlation between PET/MR parameters and molecular markers in HPC tumor tissues. Given the prognostic significance of ADC values, subsequent investigations should integrate PET/MR findings with molecular pathology data in HPC.

## Figures and Tables

**Table 1:** Clinical information of the study cohort.

Characteristic	Data (Range)
Age, median (y)	62.7 (41–77)
Tumor site	
pyriform sinus	22
posterior wall	5
postcricoid region	6
Histologic grade	
low	13
middle	19
high	1
T stage (*p*)	
T2	6
T3	16
T4	11
N stage (*p*)	
N0	7
N1	4
N2	11
N3	11
TNM stage	
II	2
III	9
IV	12
Larynx preservation	25
Recurrence	5
Death	9

**Table 2:** Accuracy and differences in clinical staging by PET/MR, CT, and MR.

	Accuracy (%)			*p*-Value		
	PET/MR	CT	MR	PET/MR vs. CT	PET/MR vs. MR	MR vs. CT
T2 (*n* = 6)	83.3	33.3	66.7	0.250	0.900	>0.999
T3 (*n* = 16)	87.5	43.8	56.3	*0.016*	0.063	0.687
T4 (*n* = 11)	100.0	81.8	81.8	>0.999	>0.999	>0.999
T (*n* = 33)	90.9	54.5	66.7	*0.001*	*0.016*	0.289
N0 (*n* = 7)	71.4	42.9	42.9	0.625	0.625	>0.999
N1 (*n* = 4)	100	25	25	>0.999	>0.999	>0.999
N2 (*n* = 11)	90.9	81.8	81.8	>0.999	>0.999	>0.999
N3 (*n* = 11)	100	81.8	81.8	>0.999	>0.999	>0.999
N (*n* = 33)	90.9	66.7	66.7	*0.021*	*0.021*	>0.999
II (*n* = 2)	100.0	50.0	50.0	>0.999	>0.999	>0.999
III (*n* = 9)	77.8	11.1	33.3	*0.031*	0.125	0.5
IV (*n* = 22)	95.5	90.9	86.4	>0.999	0.5	>0.999
Stage (*n* = 33)	90.9	66.7	69.7	*0.008*	*0.016*	>0.999

PET Positron emission tomography, MR Magnetic resonance, CT Computed tomography. Italic characters indicate *p* < 0.05.

**Table 3:** The distribution and differences of PET/MR parameters across different T stages.

	T			*p*
	T2	T3	T4	
SUVmax-T	9.2	22.0	21.8	*0.043*
SUVmean-T	5.9	13.9	13.2	0.053
TLG-T	11.9	96.6	244.2	0.074
MTV-T	2.4	5.5	15.3	*0.017*
ADCmin-T	894.3	655.0	484.7	*0.020*
ADCmean-T	1125.3	1053.2	888.2	0.051
(SUVmax/ADCmean) × 1000	11.1	21.9	25.1	*0.015*
(MTV/ADCmean) × 1000	1.9	5.9	16.3	*0.004*
(TLG/ADCmean) × 1000	13.0	93.6	256.7	*0.003*
(SUVmax/ADCmin) × 1000	39.3	46.1	60.8	*0.042*
(MTV/ADCmin) × 1000	3.9	11.4	33.9	*0.003*
(TLG/ADCmin) × 1000	40.0	196.9	505.8	*0.007*

SUV Standardized uptake value, TLG Total lesion glycolysis, MTV Metabolic tumor volume, ADC Apparent diffusion coefficient. Italic characters indicate *p* < 0.05.

**Table 4:** The distribution and differences of PET/MR parameters across different N stages.

	N				*p*
	N0	N1	N2	N3	
SUVmax-N	4.2	12.6	13.2	25.0	*0.006*
SUVmean-N	2.6	8.7	8.1	15.3	*0.007*
TLG-N	6.5	15.2	19.7	98.4	*0.000*
MTV-N	2.3	1.7	2.0	7.2	*0.000*
ADCmin-N	582.3	547.8	690.5	551.0	0.388
ADCmean-N	921.3	892.5	994.0	901.8	0.677

SUV Standardized uptake value, TLG Total lesion glycolysis, MTV Metabolic tumor volume, ADC Apparent diffusion coefficient. Italic characters indicate *p* < 0.05.

**Table 5:** The univariate and multivariate analysis of factors associated with OS.

	Univariate			Multivariate		
	HR	95%CI	*p*	HR	95%CI	*p*
T stage	/	/	0.488	/	/	/
N stage	/	/	0.694	/	/	/
TNM stage	/	/	0.863	/	/	/
Grade	/	/	0.26	/	/	/
ADCmean	0.996	0.993–0.999	*0.007*	0.995	0.992–0.998	*0.004*
ADCmin	0.997	−0.995–1.000	*0.029*	/	/	0.506
SUVmax	/	/	0.139	/	/	/
SUVmean	/	/	0.276	/	/	/
MTV	/	/	0.217	/	/	/
TLG	/	/	0.091	/	/	/
(SUVmax/ADCmean) × 1000	1.057	1.006–1.110	*0.028*			0.778
(MTV/ADCmean) × 1000	1.056	1.003–1.111	*0.037*			0.720
(TLG/ADCmean) × 1000	1.003	1.001–1.006	*0.021*	1.002	1.001–1.008	*0.022*
(SUVmax/ADCmin) × 1000	/	/	0.156	/	/	/
(MTV/ADCmin) × 1000	1.033	1.003–1.063	*0.03*	/	/	0.426
(TLG/ADCmin) × 1000	1.002	1.000–1.004	*0.021*	/	/	0.292

HR hazard ratio, SUV Standardized uptake value, TLG Total lesion glycolysis, MTV Metabolic tumor volume, ADC Apparent diffusion coefficient. Italic characters indicate *p* < 0.05.

## Data Availability

The datasets used and analyzed during the current study are available from the corresponding author on reasonable request.

## Supplementary Materials

The following supporting information can be downloaded at: https://www.mdpi.com/article/10.3390/diagnostics15172119/s1, Table S1: The distribution and differences of PET/MR parameters across different TNM stages.
