# Supplementary material for: The Diagnostic and Prognostic Value of 18F-FDG PET/MR in Hypopharyngeal Cancer
Source: Diagnostics (Basel). 2025 Aug 22;15(17):2119. doi: 10.3390/diagnostics15172119 (PMC12427711; doi:10.3390/diagnostics15172119)
Supplement: Supplementary file 1 [file diagnostics-15-02119-s001.zip › diagnostics-3779558-supplementary.pdf]

Table S1. The distribution and differences of PET/MR parameters across different TNM stages.

	TNM stage			*P*
	**II**	**III**	**IV**	
**SUVmax-T**	15.6	18.8	20.3	0.838
**SUVmean-T**	10.1	11.7	12.7	0.866
**TLG-T**	14.8	89.9	157.5	0.563
**MTV-T**	1.4	6.1	9.8	0.475
**ADCmin-T**	540.5	695.7	628.9	0.766
**ADCmean-T**	882.5	1080.3	994.8	0.421
**1000*SUVmax/ADCmean**	23.6	19.7	21.3	0.923
**1000*MTV/ADCmean**	1.9	5.7	10.1	0.438
**1000*TLG/ADCmean**	23.0	90.6	160.8	0.588
**1000*SUVmax/ADCmin**	100.5	36.2	50.1	0.303
**1000*MTV/ADCmin**	7.0	10.3	21.4	0.318
**1000*TLG/ADCmin**	100.5	174.2	326.6	0.519

SUV Standardized uptake value, *TLG* Total lesion glycolysis, *MTV* Metabolic tumor volume, *ADC* Apparent diffusion coefficient.
